# Gait retraining: altering the fingerprint of gait

**DOI:** 10.1186/1757-1146-4-S1-A5

**Published:** 2011-05-20

**Authors:** Irene Davis

**Affiliations:** 1 Dept. of Physical Medicine and Rehabilitation, Harvard Medical School, Cambridge, MA, 02138, USA

## 

Running injuries are common and are often associated with overtraining. However, it is well-accepted that these injuries are related, in part, to abnormal running mechanics. While standard interventions often result in resolution of symptoms, if the underlying mechanics are not addressed, the risk for recurrence is high. This presentation will describe a method of retraining gait patterns that requires providing realtime feedback to the runner. This feedback is slowly removed such that the runner can learn to depend upon their own internal cues and the new pattern becomes reinforced. Different types of feedback will be reviewed as well as ways to translate these methods from the lab to the clinic. The presentation will end with a case study to highlight these concepts.

